# Mercury distribution in plants and soils from the former mining area of Abbadia San Salvatore (Tuscany, central Italy)

**DOI:** 10.21203/rs.3.rs-2823040/v1

**Published:** 2023-04-19

**Authors:** Federica Meloni, Alessandro Farieri, Pablo L. Higueras, José M. Esbrí, Barbara Nisi, Jacopo Cabassi, Daniele Rappuoli, Orlando Vaselli

**Affiliations:** University of Florence; University of Florence; Instituto de Geología Aplicada, EIMIA; Complutense University of Madrid; CNR-IGG Institute of Geosciences and Earth Resources; CNR-IGG Institute of Geosciences and Earth Resources; Unione dei Comuni Amiata Val d’Orcia, Unità di Bonifica; University of Florence

**Keywords:** Mt. Amiata, Mercury, Plant uptake, Soils and Plants, Bioaccumulation factor, Foliage

## Abstract

The distribution of heavy metals in plants growing in soils from active and abandoned mining areas is of scientific significance as it allows one to recognize their ability to survive in a hostile environment and to provide useful indications for phytoremediation operations. In this work, soils developed in the former Hg-mining area of Abbadia San Salvatore (Tuscany, Central Italy) were analyzed for total, leached Hg, % of organic- and inorganic-related Hg. The dehydrogenase enzyme activity (DHA) was also measured with the aim to evaluate the status of the soil, being characterized by high Hg content. Eventually, the concentration of Hg in the different parts of the plants growing on these soils was analyzed. The soils showed Hg content up to 1068 mg kg^− 1^ and in most of them is dominated by inorganic Hg (up to 92%). The DHA concentrations were < 151 μg TPF g^− 1^day^− 1^, suggesting that the presence of Hg is not significantly affecting the enzymatic soil activity. This is also supported by the bioaccumulation factor (BF) that is < 1 in most of the studied plants. Generally speaking, the plant leaves appear to be one of the main pathways of Hg uptake, as also observed in other mining areas, e.g. Almaden (Spain), suggesting that particulate-Hg and Hg^0^ are the main forms entering the plant system, the latter derived by the GEM emitted by both the edifices hosting the roasting furnaces and the soils themselves.

## 1. Introduction

Since 2011 the world-class Hg mining district, centered in the Municipality of Abbadia San Salvatore (Tuscany, Italy) and located in the eastern part of the Mt. Amiata silicic volcanic complex (Conticelli et al., 2004, 2015; Ferrari et al., 1996; Laurenzi et al., 2015), has been undergoing remediation operations. Since the 19th century, Abbadia San Salvatore (ASS) was the site of one of the most important areas for the exploitation of cinnabar and production of liquid Hg. The ore deposit was indeed excavated, dried and roasted and, through a condensation system, liquid Hg was produced. Through the years, different furnaces were used: from those fed by wood to Spirek-Cermak, from the Gould Pacific to Nesa. It has been estimated that about 70% of the total production of Hg from the whole Mt. Amiata mines was from ASS (Cipriani & Tanelli, 1983). The mining structures of ASS produced more than 100,000 tons of liquid Hg and about 10,000 tons were dispersed into the environment (Bacci et al., 1998; Vaselli et al., 2015). Past and recent geochemical investigations (e.g. Vaselli et al., 2019 and references therein) carried out in soils, waters and air inside the mining area and surroundings have highlighted that most environmental matrices have Hg concentrations much higher than those regulated by the European legislative levels.

According to Iverfeldt (1991) and Munthe et al. (1995), the atmospheric Hg assimilation from vegetation and its transfer to soil and water via throughfall and litterfall are the main sources of Hg in the terrestrial ecosystem. According to Barquero et al. (2019), Higueras et al. (2012), and Naharro et al. (2019), plants growing on Hg-rich soils tend to uptake Hg as Hg^0^ is released from the soils to the atmosphere via stomata whereas less probable is the vehiculation of Hg via the plant roots (Naharro et al., 2019 and references therein). Concentrations of Hg-bearing organic forms in terrestrial environments are, generally speaking, at least one order of magnitude lower than those related to inorganic Hg, although the accumulation of Hg in food webs is still not clear (Bailey et al. 2002, Zhang et al., 2022). The primary warning system of alterations in terms of soil health and quality are soil enzymes, which can be considered potential bio-indicators to assess the soil health status (Datta et al., 2021 and reference therein). Among all soil enzymes, DHA (Dehydrogenase), alkaline phosphate (ALP) and urease (UR) are sensitive to both potentially toxic elements (PTEs) and minimal environmental changes (Datta et al., 2021; Elmayel et al., 2020; Gallego et al., 2021). In particular, DHA resides within all living microbial cells and, for this reason, it is considered a critical indicator of the enzymatic (and therefore, microbiological) activities of soils (Yuan and Yue, 2012). According to Mahbub et al. (2016) and Tazisong et al. (2012), scarce information is presently available about the biological functionality of Hg in plants. Nevertheless, this element has the ability to decrease enzyme activity by binding to both the protein’s -SH residues and the active sites of enzyme and protein substrate complexes, or substituting metal cofactors such as Ca or Mg and altering the structure of these com-pounds (Mahbub et al., 2016, Tazisong et al., 2012). The environmental footprint resulting from the mining activity in the Mt. Amiata and, particularly, at ASS is evidenced by numerous scientific investigations published in the last two decades (e.g. Bacci et al., 2000; Meloni et al., 2021, Rimondi et al., 2012, 2014a,b, 2015) focused on the distribution of Hg in air, soil, vegetation and aquatic compartments (e.g. Ferrara et al., 1998; Chiarantini et al., 2016, 2017; Lazzaroni et al., 2022;Vaselli et al., 2013, 2015, 2021). Previous works, conducted in the mining area of ASS (Chiarantini et al., 2016), and Almadén, (Spain) (e.g. Barquero et al., 2019), the most famous Hg-district in the world, investigated the relationship between airborne Hg (Hg^0^) and the vascular vegetation as well as the interaction between DHA and high Hg concentrations in soils, evidencing that high Hg contents in soils do not affect DHA (Campos et al., 2018). Barquero et al. (2019) and Chiarantini et al. (2016) analyzed pine trees belonging to the *Pinus Nigra* and *Pinus Pinea* families, respectively.

In this paper, the distribution of Hg between soils and the most common plants growing in the former mining area of ASS and selected parts of them (e.g. roots, trunk, leaves) is presented and discussed in order to understand at which level they are either stressed by or recalcitrant to high Hg soil content. Moreover, for the first time in this area, the possible interaction between the enzyme Dehydrogenase and Hg in the pedological cover is evaluated.

### 1.1 The Study Area: ASS mine

**Figure 1:**
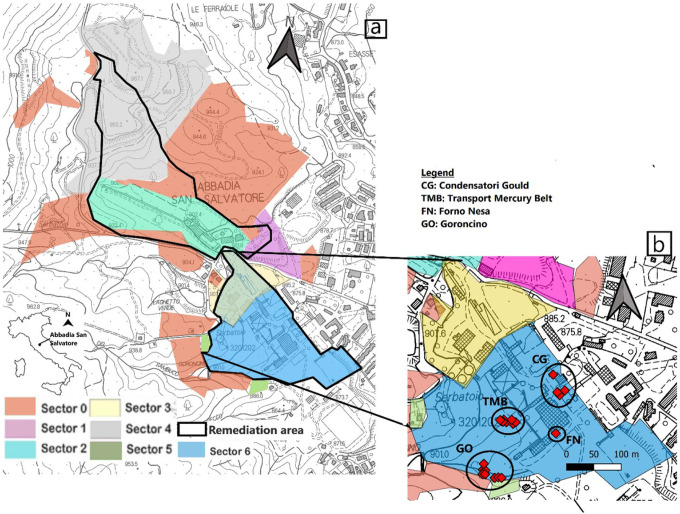
a) The reclamation area of ASS mine (modified from Vaselli et al., 2017a) scale 1:15.000. b) Inset of Sector 6 (study area) with the four sampling areas from where soils and plants were collected. The IDs of the soil and plant samples collected from each area are listed in Supplementary Material, Table S1

The ASS mining area is located in the NW part of the local urban center. The main deposits consisted of cinnabar (HgS), with smaller amounts of pyrite, marcasite, orpiment and realgar (Rimondi et al., 2015). All the exploitation was underground and the galleries reached down to −400 m below the ground level (e.g. Lazzaroni et al., 2022). Mercury production began in 1899, following the abandonment of the area for about 2,000 years since Etruscans and Romans used cinnabar as pigment (Botticelli, 2019; Fantoni et al., 2022). The old mining area includes a large wood deposit for the old furnaces, some ancient dryers, and a few tanks that were used to cool gaseous Hg as it passed through the condensers. New dryers, belt conveyor systems, and horizontal (Gould) and vertical (Nesa) furnaces were installed in the following years, along with more efficient condensation systems. In 1976, the production activity at ASS dramatically slowed down since the exploitation of Hg was not economically sustainable, since its use had become conspicuously harmful and toxic. In 1982, the whole mining plant was definitively shut down. In the 1990s, ENI (Ente Nazionale Idrocarburi) – AGIP (Azienda Generale Italiana Petroli) Unit – proposed a reclamation project to permanently close the former mining and industrial activity. In 2008, an agreement between the Municipality of ASS and the former owner of the mining concession (ENI-AGIP) was signed. The mining concession and the reclamation project was thus transferred to the public administration. The ENI project was fully revised by the local municipality and the reclamation operations were then addressed to the environmental restoration of the mining areas and buildings for museum and public utility purposes (Vaselli et al., 2019). Consequently, the whole mining concession was divided into seven different units (Fig. 1a), including the reclamation of about 65 ha (black contour in Fig. 1a): Sectors 0 and 1 constitute the sites where Hg was found at low concentrations; Sectors 2 and 3 host the miners’ and managers’ buildings, the mining equipment grounding area, the conveyor belts, and the Garibaldi shaft, and the old furnaces, dryers, and condensers, respectively; Sector 4, or ‘Le Lame’, consists of mining dump that covers a surface of 120,000 m^2^ (Meloni et al., 2021 and references therein); Sector 5 contains the armory and the watchmen’s house; Sector 6, which is the most contaminated site, hosts the main critical mining facilities, e.g. Nesa and Gould furnaces, old and new driers, condensation tubing systems, pigment preparation edifices and collection tanks of liquid Hg (Vaselli et al., 2017a). The reclamation of the ASS mine began in 2013 with the construction of the bypass channel to minimize the water-rock interaction between rainwater and Hg-contaminated soils and the removal of Eternit^®^ (a mixture of cement and asbestos fibers) from the roofs (Vaselli et al., 2015). Remediation is still ongoing and the clean-up of Sector 6 has recently begun. All soil and plant samples analyzed for this work were collected in February of 2018 from Sector 6, when the reclamation had not still started. The sampling area can be divided into: 1) Transport Mercury Belt (TMB), near the furnaces where cinnabar was roasted to extract gaseous Hg; 2) Forno Nesa (FN); 3) Goroncino (GO), where tailings from the local and other Mt. Amiata Hg-mines were stored, and 4) Gould Condensers (GC), where the gaseous Hg was cooled down after the roasting process (Fig. 1b).

## 2. Materials And Methods

Twenty-four plants (eight different species: *Castanea sativa, Sambucus nigra, Verbascum thapsus, Popolus spp., Salix spp., Acer pseudoplatanus, Robinia pseudoacacia, Cytisus scoparius*) and related soils were collected. Soils were sampled at a depth between 15 and 20 cm in order to analyze the total Hg distribution between roots and soils (Johnson et al., 2005). Two years before the sampling date, a complete vegetation clearcutting was carried out. Thus, the age of the sampled plants was known. It was decided to divide the plant samples into bark, internal and external roots bark and internal trunks, medulla part (when the trunk or root presented it), and foliage. Table S1 reported the geographic coordinates in WGS84-UTM 32N, the soil IDs, the sampling location, the Latin name of the sampled plants and the parts into which each plant was divided and analyzed. For each plant sample, the soil particles were brushed off manually. Subsequently, the plant samples were washed in an ultrasonic bath until the MilliQ water was clean. Then, they were heated in an oven for at least three days at 35°C. Eventually, each plant portion was ground into small pieces using a coffee grinder.

The soil samples were stored in an oven at a temperature of 35°C (to prevent any Hg^0^ loss) until they were dried, and then sieved at 2 mm. A representative aliquot of the < 2 mm samples (about 100 g) was pulverized in a planetary mill (Pulverisette 5) equipped with agate mortars and balls. The pH values for each soil were determined following the UNI_EN 15933:2012 norm using a multi-probe Hanna HI98194.

Mercury concentrations in soils and plants were determined by a Lumex RA 915+ (Atomic Absorption Spectrometry with Zeeman effect) instrument (Sholupov et al., 2004), equipped with a Pyro-915 + device. Additionally, the percentage of Hg related to the organic and inorganic fractions was also estimated (Rumayor et al., 2016). In fact, it must be taken into account that biological samples burn at 275–290°C. At this temperature interval, all organic Hg compounds decompose and release Hg.

The analysis of DHA was aimed at quantifying the amount of organic activity in soils. A mixture consisting of 0.15 mL of distilled water, 0.015 g of CaCO_3_, and 0.25 mL of 3% (m/V) of Triphenyl-Tetrazolium-Chloride (TTC, C_19_H_15_ClN_4_) was prepared and it was left reacting with 1.5 g of soil. The samples were then introduced in an incubator at 37°C for 24h (Casida et al., 1964; Montejo et al., 2012) and eventually, cooled in a freezer for 10 min to stop the reaction. Subsequently, 5 mL of methanol were added to extract the formed TTC. The colored extract was measured at 485 nm by molecular spectrophotometry. All these analyses were carried out at the IGeA Laboratories (Instituto de Geología Aplicada, University of Castilla La Mancha) in Almadén (Ciudad Real, Spain).

The leachable Hg was determined by ICP-MS using the USEPA 1312 method (USEPA, 1994) at the CSA Laboratories in Rimini (Italy), in order to simulate the fraction of soluble Hg in acid mine drainage conditions. This extraction entails mixing 5 g of soil sample with 100 mL of the EPA solution. A 60/40 combination of H_2_SO_4_ and HNO_3_ was added to 2 L of distilled water to create the EPA solution (pH of 4.5 ± 0.05). After mixing the soil with the EPA solution, the samples were heated in a stirrer thermostatic bath for 18 hours at 30 revolutions per minute. Water was added to maintain a constant temperature of 25°C. After 18 hours, the samples were filtered using glass fiber filters with a 0.45 μm pore size.

### 2.1 Hg Bioaccumulation Factor

According to Campos et al. (2018), the Bioaccumulation Factor reflects the bioavailability of Hg in plants. Bioaccumulation Factor (BF) is a parameter used to measure the transfer capacity of PTEs from soil to plant (Wang et al., 2016). In this study, BF was calculated as the ratio between the Hg concentration in the different parts of the plants and the soil leachable Hg content (Eq. 1):

Eq. 1 $$BF=\frac{Hg_{p}}{Hg_{SL}}$$

where Hg_p_ is the Hg concentration in the selected part of each plant whilst Hg_SL_ is the leachable Hg content recalculated to the amount of leached soil. Differently to other elements, in this case BF expresses the uptake of the element via indirect ways. Basically, it can be referred to the Hg uptake and bioaccumulation in the different analyzed plant parts.

## 3. Results

### 3.1 Hg concentration and DHA in top-soils

The main descriptive statistics of the pH, total Hg, leached Hg, soil leachable Hg, % Hg inorganic, % Hg organic, and DHA values in the studied soils (e.g. number of observations, minimum, maximum, mean, median, standard deviation) are summarized in Table 1. The full dataset is reported in Table S2 (Supplementary Material).

The pH values of ASS mining soils are mostly alkaline. Total Hg in soils reached an average value of 462 mg kg^− 1^, with a minimum and maximum concentrations of 2 and 1068 mg kg^− 1^, respectively. The minimum concentration of mercury in the leached soil was below the instrumental detection limit (0.1 μg L^− 1^, sample ASS2), whereas the maximum concentration was 20.4 μg L^− 1^ (ASS14). Soil leachable Hg present a maximum value of 8.56 mg kg^− 1^, whereas the minimum value of the ASS2 soil sample could not be determined. The percentage of inorganic and organic Hg in soils, with the exception of sample ASS1 (where it was not possible to measure the relative percentage) had maximum values of 93.9% and 73.5%, respectively. Regarding the DHA, only the sample ASS2 had concentration < 1 μg TPF g^− 1^day^− 1^, whereas the maximum value was 166.0 μg TPF g^− 1^day^− 1^ (ASS8b).

### 3.2 Hg in plants

The minimum, maximum of BF values and minimum, maximum and median of Hg concentrations in the eight types of plants, without distinguishing the different plant portions, are summarized in Table 2. All data are listed in Table S3 (Supplementary Material).

According to Table 2, *Verbascum thapsus* is the plant where the highest Hg concentration (54.54 mg kg^− 1^) was measured, whereas the lowest content pertained to *Acer pseudoplatanus* (3.13 mg kg^− 1^). As far as BF is concerned, *Sambucus nigra* and *Verbascum thapsus* are the two plant species able to accumulate the highest amount of Hg (max. values BF: 0.93 and 0.64, respectively). The remaining plants show a maximum BF value of < 0.60 while that of minimum was < 0.001 for *Castanea Sativa, Sambucus nigra, Acer pseudoplatanus and Robina pseudoacacia*.

## 4. Discussion

As far as the total Hg distribution in soils is concerned, the GC zone results to be the area with the highest concentration of Hg, followed by TMB > GO > FN (Fig. 1b). No significant correlation between total and leached Hg is observed, indicating that Hg is heterogeneously distributed in the investigated soils, being likely related to different sources. In contrast to Campos et al. (2018), who reported a correlation between total Hg and soil leached Hg of 0.79, and a correlation between soil leached Hg and humic acid Hg of 0.65, in this work soil leached Hg did not present any correlation between the Hg species analyzed. The top-soils are indeed affected by the presence of anthropogenic materials. In the past, in some portions of the former mining area, post-roasting and anthropic man-made (e.g. bricks, tiles, fragments of concrete) materials were used to fill a small paleo-valley positioned in front of the edifice hosting the Gould and Nesa furnaces (Fig. 1b) (Vaselli et al., 2015).

**Figure 2:**
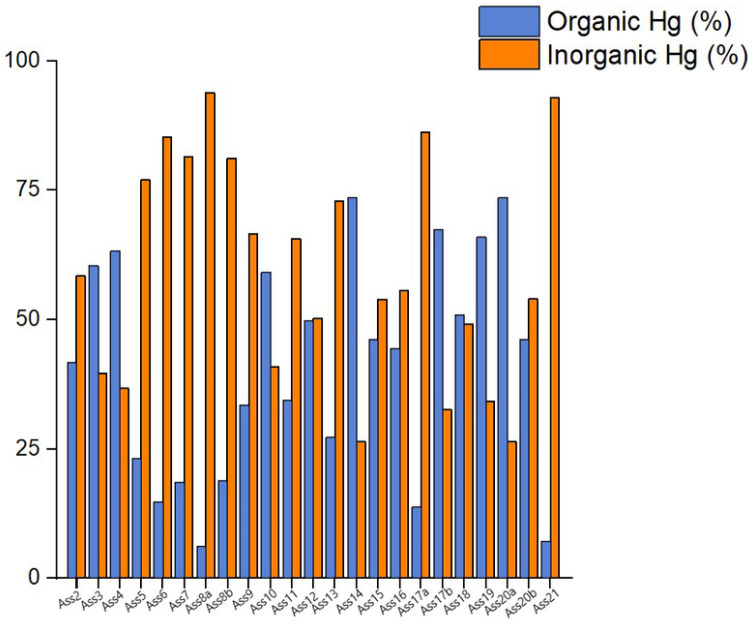
Bar chart plot of organic and inorganic Hg (in %) in the soil samples

The thermal speciation data evidenced that most Hg is inorganic although eight top-soils (i.e., ASS3, ASS4, ASS10, ASS14, ASS17b, ASS18, ASS19, and ASS20a) have a percentage of organic-related Hg that prevails over that related to inorganic Hg, as evidenced in the bar plot chart of Fig. 2.

Figure 2 Bar chart plot of organic and inorganic Hg (in %) in the soil samples

**Figure 3:**
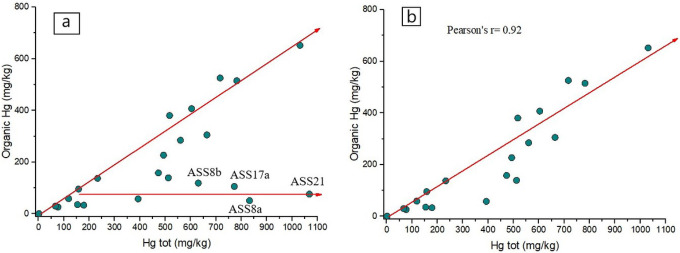
Binary diagram between organic-related Hg vs. Total Hg (in mg kg^−1^) with (a) and without sampled ASS8a,b, ASS17a, ASS21 (b). The organic Hg concentrations are those computed by thermal speciation

**Figure 4:**
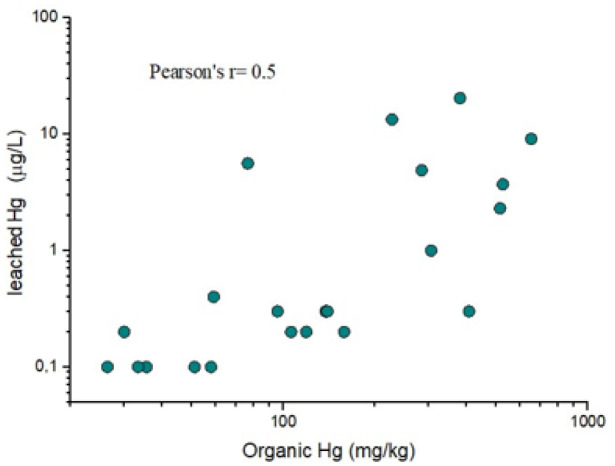
Binary diagram between inorganic-related Hg vs. Total Hg (in mg kg^−1^). The inorganic Hg concentrations are those computed by thermal speciation

In this study, as well as in the Almadenejos metallurgical precinct (Almadén, Spain) by Campos et al. (2018), a positive correlation was found between total Hg and that corresponding to the fractions identified by thermal speciation, i.e. organic Hg and inorganic Hg (Fig. 3a,b and Fig. 4). In Fig. 3a, two distinct trends can be observed. The first one corresponds to a positive correlation between total Hg and organic Hg whereas the second trend is mainly delineated by four samples (ASS8a, ASS8b, ASS17a and ASS21), which are characterized by an increasing concentration of computed inorganic Hg (up to > 80%) whilst that of organic Hg maintains almost unchanged. We can hypothesize that these soil samples are possibly indicating the presence of higher contents of residual mining materials. When these four samples are not considered, the correlation between the two parameters significantly increases as a Pearson coefficient of 0.92 (Fig. 3b) was computed. A similar positive correlation (r = 0.92) is also obtained when total Hg is plotted *vs*. inorganic-related Hg (Fig. 4). This interdependence is likely indicating that the fractionation of Hg compounds in the soils of the ASS mine is a distinct process unaffected by the relative position of the samples, the amount of organic matter present, or the activity due to enzymatic processes, as also reported by Campos et al. (2018). It is be noticed that the soil samples located in GO (Fig. 1b and Table S1) are more enriched in organic-related Hg with respect to those collected close to the mining facilities, suggesting that the proximity to the machineries to produce liquid Hg affected the soil matrix.

**Figure 5:**
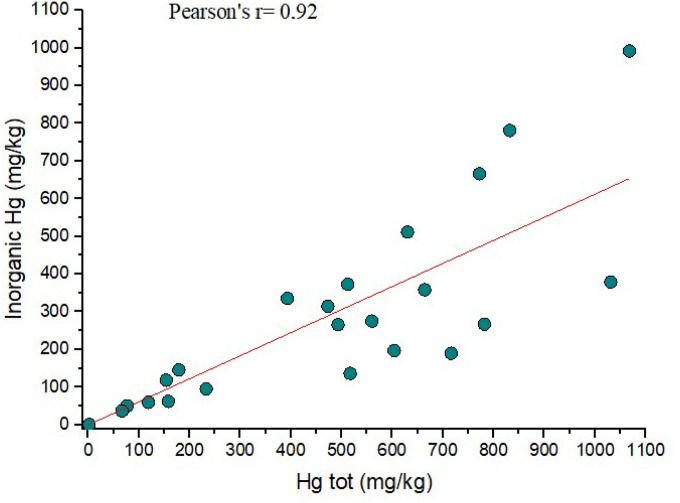
Log-scale binary diagram between leached (in μg L^−1^) and organic-related Hg. The organic Hg concentrations are those computed by thermal speciation

In addition, a second, weaker correlation (r = 0.5) between leachable Hg (in μg L^− 1^) and organic-related Hg (in mg kg^− 1^) is reported in Fig. 5. According to Campos et al. (2018), the most labile species of Hg are those containing organic Hg. However, it is to be pointed out that the high concentration of leached Hg (up to 20 μg L^− 1^) can also be released by solid phases and not necessarily only related to organic Hg.

Figure 4 Binary diagram between inorganic-related Hg *vs*. Total Hg (in mg kg^− 1^). The inorganic Hg concentrations are those computed by thermal speciation

The measured DHA contents have an average value of 53.7 μg TPF g^− 1^day^− 1^. This value is markedly lower than that measured by Campos et al. (2018) in the Almadenejos soils and approaches that reported by Hinojosa et al. (2004) for soils polluted by heavy metals (average: 70 μg TPF g^− 1^day^− 1^).

**Figure 6:**
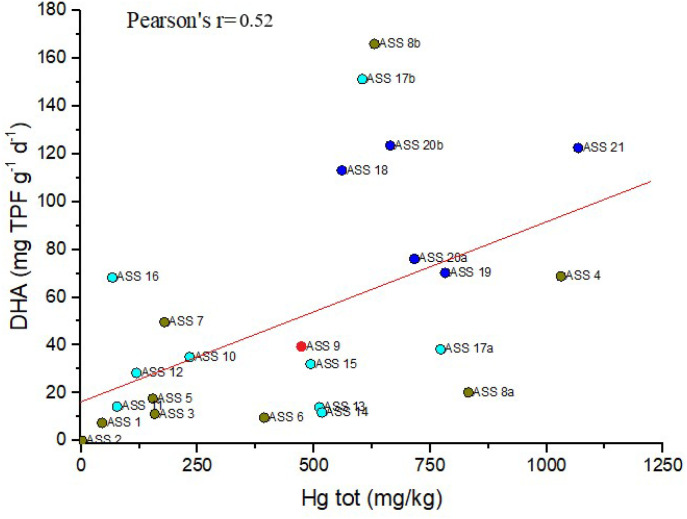
Scatterplot of DHA (mg TPF g^−1^d^−1^) vs. Hg (mg kg^−1^) in the soils from the ASS mining area. Blue circles: samples from CG, red circles: samples from FN, cyan circle: samples from GO and dark yellow circle: samples from TMB

The concentration of DHA in the soils from the mining and production area appears to be even lower than those measured by Hinojosa et al. (2004) in the reclaimed area of the Aznalcollar mine (SW Spain). According to Pan and Yu (2011), the presence of PTEs in soils can have negative effects on the enzymatic activity, affecting either the enzyme-substrate complexation or the structure of the amino acids. In this case, the total Hg *vs*. DHA enzyme diagram (Fig. 6) shows a poor correlation (Pearson correlation r = 0.52, p < 0.05) with scatter distribution between the two parameters, suggesting that the presence of Hg, independently by its speciation, is not able to affect the microbial activity in the ASS soils, similarly to what observed by Campos et al. (2018) for the Hg-rich soils from Almadenejos.

Figure 6 Scatterplot of DHA (mg TPF g^− 1^d^− 1^) *vs*. Hg (mg kg^− 1^) in the soils from the ASS mining area. Blue circles: samples from CG, red circles: samples from FN, cyan circle: samples from GO and dark yellow circle: samples from TMB

Considering the metals and metalloids usually found in the AAS ore deposits and the surrounding Hg-mining areas (Rimondi et al., 2014a), the analytical spectrum should be enlarged to evidence whether, the enzymatic activity may be jeopardized by other PTEs (e.g. As and Sb).

### 4.1. Hg and BF in plants

**Figure 7:**
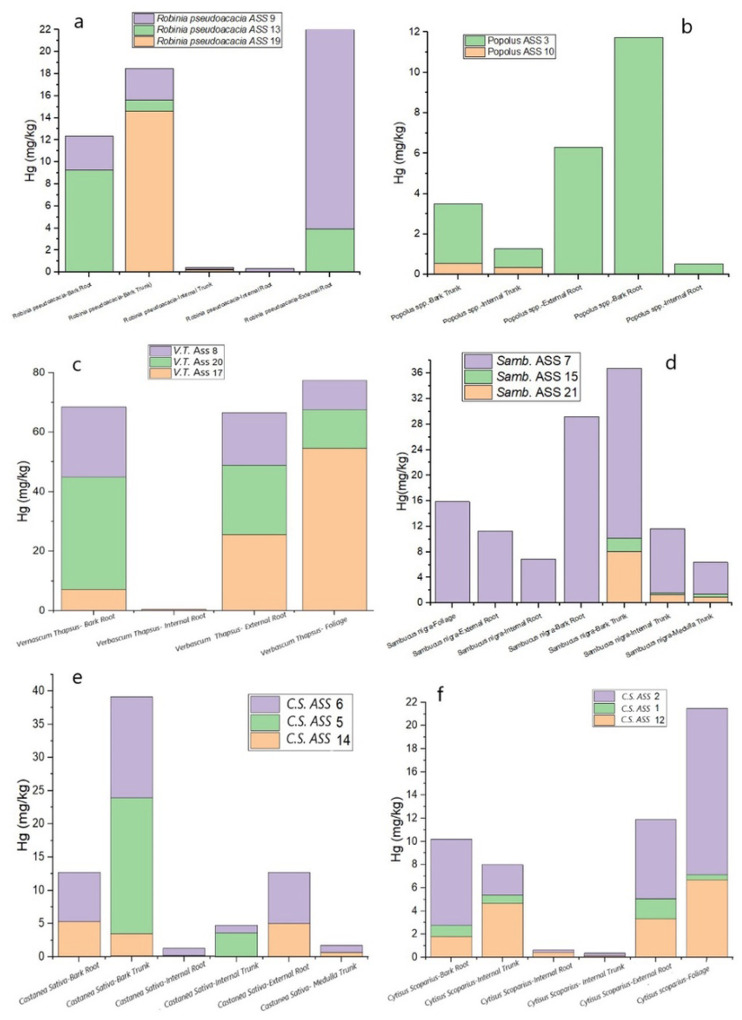
Bar graphs of the Hg amount in each analyzed portion of *Robinia pseudoacacia* (a), *Popolus spp*. (b), *Verbascum thapsus* (c) *Sambucus nigra* (d), *Castanea sativa* (e), and *Cytisus scoparius* (f)

The bar graphs in Fig. 7 depict the Hg distribution in each portion of the sampled plants, except for *Acer pseudoplatanus* and *Salix spp*., for which only one sample (bark trunk and root, respectively) was collected. *Robinia pseudoacacia, Sambucus nigra, Castanea sativa* and *Popolus spp*. are characterized by the highest Hg concentrations in the roots, as well as *Salix spp*. while the bark trunk of *Acer* is enriched in Hg. Different is the behavior of *Cytisus scoparius* and *Verbascum thapsus* as Hg is found in high contents in the foliage. Notably, is the fact that the highest Hg concentrations are related to the leaves of *Cytisus scoparius*, located in the TMB zone, where the gaseous elemental Hg in the atmosphere were found almost constantly up to 50,000 ng m^− 3^ or even higher (Vaselli et al., 2013).

This is likely related to the fact that the leaf system is one of the main pathways of Hg uptake due to both dry deposition, as also suggested by Chiarantini et al. (2016) and Campos et al. (2018), as well as gaseous Hg from Hg-rich environments (such as that recorded in the air nearby the mining machineries and furnaces) and diffuse Hg from soil, although no Hg flux measurements are presently available.

The high Hg concentrations detected in the leaves of *Verbascum thapsus* (related to ASS 17 soil), collected from GO (Fig. 1b), can be explained by the main winds at ASS that blow from NNE/NE (https://www.meteoblue.com/it/tempo/historyclimate/climatemodelled/abbadia-san-salvatore_italia_3183581), thus, favoring the deposition of the Hg-rich atmospheric particulate and atmospheric Hg from TMB to GO (Fig. 1b). Therefore, according to the investigation on the different parts of plants analyzed in this study (Fig. 7), foliage is likely the main mechanism of Hg-uptake that can be invoked for the ASS plants, thus confirming previous investigations, e.g. Naharro et al., (2019). Occasionally, roots seem to play a role in the Hg-uptake. However, further analyses on the leaf apparatus for those plants where the foliage was not collected are necessary.

**Figure 8:**
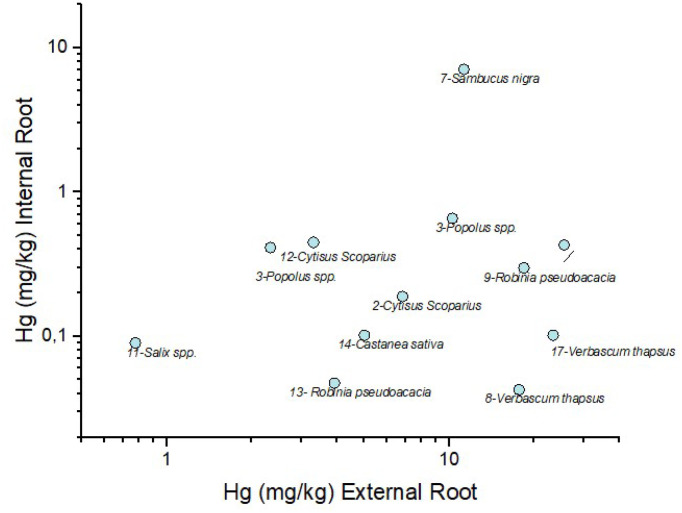
Scatterplot of Hg (mg kg^−1^) in external root vs. Hg (mg kg^−1^) in internal root of the plant samples in bi-logarithmic scale. The numbers before the plant name correspond to the related soil (see Table S1)

The distribution of Hg between external and internal roots is reported in Fig. 8. All the studied root samples indicate that Hg concentration increases, as expected, in the external roots, with the exception of *Sambucus nigra*, showing an external root/internal ratio of about 1.7, i.e. more than one order of magnitude lower that those recorded for those samples characterized by Hg content > 20 mg kg^− 1^. To the best of our knowledge, few are the studies related to the partitioning of Hg between internal and external roots and this calls for more detailed investigations.

According to Hussain et al. (2022), BF is a partitioning coefficient that mimics the ability of plants to absorb PTEs and, in this study, it was applied to the concentration of Hg in each part of the analyzed plants and that related to the soil leachable Hg. The BF values are highly variable when the different plant sectors are considered (Table S3). All BF values are < 1 (Table S3), but BF > 0.6 values correspond to the bark trunk and bark root of *Sambucus nigra*, and the leaves of *Verbascum thapsus* (0.63, 0.93, and 0.65 respectively). The BF values in the leaves of *Verbasum thapsus* seem to confirm that the leaves are likely the main path of Hg-uptake by plants. On the other hand, the relatively high BF values measured in the bark trunk and bark roots of *Sambucus nigra* are possibly due to the difficulty in efficiently and completely removing all the soil-related particles during cleaning. This means that the concentration of mercury in the outermost part of roots and trunk is likely affected by the presence of soil material.

## 5. Conclusions

In this study, the distribution of Hg in soils and plants growing in the former mining area of Abbadia San Salvatore was investigated and, to best of our knowledge, DHA concentrations and the BF values were determined for the very first time in one of the most important Hg sites worldwide. The content of Hg in the soils located in the highly Hg contaminated Sector 6 is heterogeneously distributed in the four, high Hg-contaminated, areas where the plants were collected (Fig. 1b). The highest concentrations were measured close to the edifices hosting the Nesa and Gould furnaces, since here, when the mining works were active, mine tailings were used to fill a small paleo-valley. Thermal speciation allowed to recognize that inorganic Hg, presumably associated with cinnabar, with the exception of eight soils, was the prevailing species over the organic component. The low DHA concentrations, in agreement with Hinojosa et al. (2004), indicates that the area is likely contaminated by PTEs, although investigations are required to evidence their presence. However, the poor, though positive, correlation between total Hg and DHA shows that the Hg compounds do not affect the enzymatic action of DHA and do not inhibit but, conversely, enhance microbial activity. Mercury concentrations in different portions of the analyzed plants show that the main pathway of mercury uptake is the leaf system. For those samples where the foliage was not analyzed, roots are likely playing an important role in the uptake of Hg. However, more detailed investigations are needed to fully understand i) the role played by roots when developed in a Hg-rich pedological environment and ii) the partitioning of Hg between external and internal roots. The Hg-BF is < 1 in all samples, indicating a weak translocation of mercury from the soil to the plant. In order to avoid possible errors in computing BF calculation, more careful and repeated washing of the different parts of the plants (especially those parts that are most in contact with the soil) is recommended. According to this study, phytoremediation projects should take into account the ability of *Sambucus nigra* to uptake Hg. A pilot site consisting of a *Sambucus nigra* plantation, positioned in a secure disposal location within the remediation area, could be established to verify whether the Hg removal is effective. Nevertheless, it can be recommended that more indigenous plants should be thoroughly analyzed to verify whether other plant species may have a stronger Hg adsorption capacity than that of *Sambucus nigra*.

## Figures and Tables

**Table 1:** Number of observations (N. obs.), minimum, maximum, average, median, and standard deviation (SD) of pH, Total Hg (in mg kg^− 1^), leached Hg (in μg L^− 1^), soil leachable Hg (in mg kg^− 1^), inorganic Hg (in %), organic Hg (in %), and DHA (in μg TPF g^− 1^day^− 1^). n.d.: not detected. The full data are reported in Supplementary Material-Table S2.

	N. obs.	Minimum	Maximum	Average	Median	SD
pH	24	7.9	8.8	8.5	8.5	0.2
Total Hg	24	2	1068	462	503	318
Leached Hg	24	< 0.1	20	3	0.3	5
Soil Leachable Hg	24	n.d.	8.56	1.15	0.12	2.12
Inorganic Hg	24	n.d.	94	59	56	22
Organic Hg	24	n.d.	74	41	44	22
DHA	24	< 1	166	54	37	49

**Table 2:** Minimum (min), maximum (max) of Bioaccumulation Factor (BF) values and minimum (min), maximum (max), and median of Hg concentrations (mg kg^− 1^) in the eight plants species considered in the present work. No distinction based on single plant parts is done (see below); n.a.: not available. The full data are shown in Table S3 (Supplementary Material)

Plants	BF		Hg(mgkg^−*1*^)	
	Min.	Max	Min	Max
*Castanea sativa*	< 0.001	0.56	0.073	23.74
*Sambucus nigra*	< 0.001	0.93	0.263	39.42
*Verbascum thapsus*	0.001	0.64	0.043	54.54
*Popolus spp*.	0.002	0.13	0.279	16.86
*Salix spp*.	0.002	0.14	0.12	6.17
*Acer pseudopla tan us*	< 0.001	0.008	0.09	3.13
*Robinia pseudoacacia*	< 0.001	0.12	0.05	18.4

## References

[1] Bacci E., Gaggi C., Lanzillotti E., Ferrozzi S., & Valli L. (2000). Geothermal power plants at Mt. Amiata (Tuscany-Italy): mercury and hydrogen sulphide deposition revealed by vegetation. Chemosphere, 40, 907–911. 10718585 10.1016/s0045-6535(99)00458-0

[2] Bailey E.A., Grey J.E., & Theodorakos P.M. (2002). Mercury in vegetation and soils at abandoned mercury mines in southwestern Alaska, USA. GEEA, 2, 275–285. 10.1144/1467-787302-03 11032113

[3] Barquero J. I., Rojas S., Esbrí J. M., García-Noguero E. M., & Higueras P. (2019.) Factors Influencing Mercury Uptake by Leaves of Stone Pine (Pinus Pinea L.) in Almadén (Central Spain). Environmental Science and Pollution Research, 26, 3129–3137. https://doi.or/10.1007/s11356-017-0446-8 29090438 10.1007/s11356-017-0446-8

[4] Botticelli M. (2019). Archaeometric investigations on red pigments: the provenance of cinnabar and the discrimination of synthetic and natural ochres. PhD Thesis, University of Rome “La Sapienza”, (pp. 271).

[5] Campos J.A., Esbrí J.M., Madrid M. M., Naharro R., Peco J., García-Noguero E.M., Amorós J.A., Moreno M. M., & Higueras P. (2018). Does Mercury Presence in Soils Promote Their Microbial Activity? The Almadenejos Case (Almadén Mercury Mining District, Spain). Chemosphere, 201,799–806. 10.1016/j.chemosphere.2018.02.163 29550574

[6] Casida L.E., Klein D.A., & Santoro T. (1964). Soil dehydrogenase activity. Soil of Science, 98, 371–376

[7] Chiarantini L., Rimondi V., Benvenuti M., Beutel M. W., Costagliola P., Gonnelli C., Lattanzi P., & Paolieri M., (2016). Black Pine (Pinus Nigra) Barks as Biomonitors of Airborne Mercury Pollution. Science of The Total Environmental, 569–570, 105–113. 10.1016/j.scitotenv.2016.06.029. 27341111

[8] Chiarantini L., Rimondi V., Bardelli F., Benvenuti M., Cosio C., Coatagliola P., Di Benedetto F., Lattanzi P., & Sarret G. (2017). Mercury speciation in Pinus nigra barks from Monte Amiata (Italy): An X-ray absorption spectroscopy study. Environmental Pollution., 227, 83–88. 10.1016/j.envpol.2017.04.038. 28458249

[9] Cipriani C., & Tanelli G. (1983). Risorse minerarie ed industria estrattiva in Toscana. Note storiche ed economiche. Atti Mem. Accademia Toscana di Scienze e Lettettere “La Colombaria”, 28, 241–283. (In Italian)

[10] Conticelli S., Melluso L., Perini G., Avanzinelli R., & Boari E. (2004). Petrologic, Geochemical and Isotopic characteristics of potassic and ultrapotassic magmatism in Central-Southern Italy: Inferences on its genesis and on the nature of mantle sources. Periodico di Mineraleralogia, 73, 135–164.

[11] Conticelli S., Boari E., Burlamacchi L., Cifelli F., Moscardi F., Laurenzi M.A., Ferrari Pedraglio L., Francalanci L., Benvenuti M.G., Braschi E., & Manetti P. (2015). Geochemistry and Sr-Nd-Pb Isotopes of Monte Amiata Volcano, Central Italy: Evidence for magma mixing between high-K calc-alkaline and leucititic mantle-derived magmas. Italian Journal of Geoscience, 134, 266–290. 10.3301/IJG.2015.12

[12] Datta A., Gujre N., Gupta D., Agnihotri R., & Mitra S. (2021). Application of enzymes as a diagnostic tool for soils as affected by municipal solid wastes. Journal of Environmental Management, 286, 112169. 10.1016/j.jenvman.2021.112169 33621849

[13] Elmayel I., Esbrí J.M., García-Ordiales E., Bouzid J., Garcia-Noguero E.M., Elouaer Z., Campos J.A., & Higueras P. (2020a). Biogeochemical assessment of affection by mining activity in the mining area of Jebal Trozza mine, Central Tunisia. Environmental Geochemistry and Health,42, 3529–3542. 10.1007/s10653-020-00595-2 32399635

[14] Fantoni R., Lazic V., Colao F., Almaviva S., & Puiu A. (2022). Caracterización del color rojo en varios frescos y pinturas romanas in situ y remotas mediante espectroscopías LIBS, LIF y Raman. Ge-Conservacion, 21(1), 257–269. 10.37558/gec.v21i1.1117

[15] Ferrara R., Mazzolai B., Edner H., Svenberg S., & Wallinder E. (1998). Atmospheric mercury sources in the Mt. Amiata area, Italy. Science of The Total Environmental, 213, 13–23.

[16] Ferrari L., Conticelli S., Burlamacchi L., & Manetti P. (1996). New geologic and volcanological data on the Mt. Amiata silicic complex. Acta Vulcanologica, 8, 41–56

[17] Gallego S., Esbrí J.M., Campos J.A., Peco J.D., Martin-Laurent F., & Higueras P. (2021). Microbial diversity and activity assessment in a 100-year-old lead mine. Journal of Hazardous Material, 410,124618. 10.1016/j.jhazmat.2020.124618 33250311

[18] Higueras P., Amorós J.A., Esbrí J.M., García-Navarro F.J., Pérez de los Reyes C., & Moreno G. (2012). Time and space variations in mercury and other trace element contents in olive tree leaves from the Almadén Hg-mining district. Journal of Geochemical Exploration, 123, 143–151. 10.1016/j.gexplo.2012.04.012

[19] Hinojosa M.B., Carreira J.A., Garcia-Ruiz R., & Dick R.P. (2004). Soil moisture pretreatment effects on enzyme activities as indicators of heavy metal-contaminated and reclaimed soils. Soil Biology and Biochemistry, 36, 1559–1568. 10.1016/j.soilbio.2004.07.003

[20] Hussain S., Yang J., Hussain J., Sattar A., Ullah S., Hussain I., Rahman S.U., Zandi P., Xia X., & Zhang L. (2022). Mercury fractionation, bioavailability, and the major factor predicting its transfer and accumulation in soil-wheat systems. Science of The Total Environmental, 847, 157432 10.1016/j.scitotenv.2022.157432 35853525

[21] Iverfeldt Å. (1991). Occurrence and turnover of atmospheric mercury over the Nordic countries. Water Air & Soil Pollution, 56, 251–265. 10.1080/10934529.2012.665000

[22] Johnson D. L., Domier J. E. J., & Johnson D. N. (2005). Reflections on the Nature of Soil and Its Biomantle. Annals of American Association of Geographers 95, 1, 11–31 10.1111/j.1467-8306.2005.00448.x

[23] Laurenzi M.A., Braschi E., Casalini M., & Conticelli S. (2015). New 40 Ar-39Ar dating and revision of the geochronology of the Monte Amiata Volcano, Central Italy. Italian Journal of Geoscience, 134, 255–265. 10.3301/IJG.2015.11

[24] Lazzaroni M., Vetuschi Zuccolini M., Nisi B., Cabassi J., Caliro S., Rappuoli D., & Vaselli O. (2022). Mercury and Arsenic Discharge from Circumneutral Waters Associated with the Former Mining Area of Abbadia San Salvatore (Tuscany, Central Italy). International Journal of Environmental Research and Public Health, 19, 5131. 10.3390/ijerph19095131 35564526 PMC9103097

[25] Mahbub K.R., Krishnan K., Megharai M., & Naidu R. (2016). Mercury inhibits soil enzyme activity in a Lower Concentration than the Guideline Value. Bullettin of Environmental Contamination and Toxicology, 96, 76–82. 10.1007/s00128-015-1664-8 26438177

[26] Meloni F., Montegrossi G., Lazzaroni M., Rappuoli D., Nisi B., & Vaselli O. (2021). Total and Leached Arsenic, Mercury and Antimony in the Mining Waste Dumping Area of Abbadia San Salvatore (Mt. Amiata, Central Italy). Applied Science, 11, 7893. 10.3390/app11177893

[27] Meteoblue. Available online: https://www.meteoblue.com/it/tempo/historyclimate/climatemodelled/abbadia-san-salvatore_italia_3183581 (11 December 2022).

[28] Montejo M.M., Torres C.P., Martínez A., Tenorio J.A., Cruz M.R., Ramos F.R., & Cuevas M.C. (2012). Técnicas para el análisis de actividad enzimática en suelos. In: Métodos ecotoxicológicos para la evaluación de suelos contaminados con hidrocarburos, Cuevas M.C., Espinosa G., Ilizaliturri C., Mendoza A. (Eds.), Istituto Nacional de Ecologia (NIE), Mexico,(1th ed., pp. 19–47).

[29] Munthe J., Hultberg H., & Iverfeldt Å. (1995). Mechanisms of deposition of methylmercury and mercury to coniferous forests. Water Air & Soil Pollution, 80, 363–371. 10.1007/BF01189686

[30] Naharro R., Esbrí J.M., Amorós J.A., García-Navarro F.J., & Higueras P. (2019). Assessment of mercury uptake routes at the soil-plant-atmosphere interface. GEEA, 19(2), 146–154. 10.1144/geochem2018-019

[31] Pan J., Yu L., 2011. Effects of Cd or/and Pb on soil enzyme activities and microbial community structure. Ecol. Eng. 37, 1889–1894. 10.1016/j.ecoleng.2011.07.002

[32] Rimondi V., Grey J.E., Costagliola P., Vaselli O., & Lattanzi P. (2012). Concentration, distribution, and translocation of mercury and methylmercury in mine-waste, sediment, soil, water, and fish collected near the Abbadia San Salvatore mercury mine, Mt. Amiata district, Italy. Science of The Total Environmental, 414, 318–327 10.1016/j.scitotenv.2011.10.065 22169390

[33] Rimondi V., Costagliola P., Gray J.E., Lattanzi P., Nannucci M., Paolieri M., & Salvadori A. (2014a). Mass loads of dissolved and particulate mercury and other trace elements in the Mt. Amiata mining district, Southern Tuscany (Italy). Environmental Science and Pollution Research, 21, 5575–5585. 10.1007/s11356-013-2476-1 24414225

[34] Rimondi V., Bardelli F., Benvenuti M., Costagliola P., Grey J.E., & Lattanzi P. (2014b). Mercury speciation in the Mt. Amiata mining district (Italy): interplay between urban activities and mercury contamination. Chemical Geology, 380, 110–118 10.1016/j.chemgeo.2014.04.023

[35] Rimondi V., Chiaranti L., Lattanzi P., Benvenuti M., Beutel M., Colica A., Costagliola P., Di Bendetto F., Gabbani G., Gray J.E., Pandeli E., Pattelli G., Paolieri M., & Ruggieri G. (2015). Metallogeny, explotation and environmental impact of the Mt. Amiata mercury ore district (Southern Tuscany, Italy). Italian Journal of Geoscience, 134, 323–336 10.3301/IJG.2015.02

[36] Rumayor M., Lopez-Anton M.A., Díaz-Somoano M., Maroto-Valer M.M., Richard J., Biester H., Martínez-Tarazona M.R. (2016). A comparison of devices using thermal desorption for mercury Speciation in solids. Talanta, 150, 272–277. 10.1016/j.talanta.2015.12.058 26838408

[37] Sholupov S., Pogarev S., Ryzhov V., Mashyanov N., & Stroganov A. (2004). Zeeman atomic absorption spectrometer RA-915 for direct determination of mercury in air and complex matrix samples. Fuel Processing Technology, 85 (6–7), 473–485.

[38] Tazisong I.A., Senwo Z.N., & Williams M.I. (2012). Mercury speciation and effects on soil microbial activities. Journal of Environmental Science and Health, 47, 854–862 10.1080/10934529.2012.665000 22423992

[39] Vaselli O., Higueras P., Nisi B., Esbrí J.M., Cabassi J., Martinez-Coronado A., Tassi F., & Rappuoli D. (2013). Distribution of Gaseous Hg in the Mercury Mining District of Mt. Amiata (Central Italy): A Geochemical Survey Prior the Reclamation Project. Environmental Research, 125, 179–87. 10.1016/j.envres.2012.12.010 23477568

[40] Vaselli O., Nisi B., Rappuoli D., Bianchi F., Cabassi J., Venturi S., Tassi F., & Raco B. (2015). Geochemical characterization of the ground waters from the former Hg-mining area of Abbadia San Salvatore (Mt. Amiata, central Italy): Criticalities and perspectives for the reclamation process. Italianl Journal of Geoscience, 134, 304–322. 10.3301/IJG.2015.03

[41] Vaselli O., Nisi B., Rappuoli D., Cabssi J., & Tassi F. (2017a). Gaseous Elemental Mercury and Total and Leached Mercury in Building Material from the Former Hg-Mining Area of Abbadia San Salvatore (Central Italy). International Journal of Environmental Research and Public Health, 14, 425. 10.3390/ijerph14040425 28420130 PMC5409626

[42] Vaselli O., Rappuoli D., Bianchi F., Nisi B., Niccolini M., Esposito A., Cabassi J., Giannini L., & Tassi F. (2019). One hundred years of mercury exploitation at the mining area of Abbadia San Salvatore (Mt. Amiata, Central Italy): A methodological approach for a complex reclamation activity before the establishment of a new mining park. El patrimonio geológico y minero. Identidad y motor de desarrollo. (pp. 1109–1126). Instituto Geológico y Minero de España.

[43] Vaselli O., Lazzaroni M., Nisi B., Cabassi J., Tassi F., Rappuoli D., & Meloni F. (2021). Discontinuous Geochemical Monitoring of the Galleria Italia Circumneutral Waters (Former Hg-Mining Area of Abbadia San Salvatore, Tuscany, Central Italy) Feeding the Fosso Della Chiusa Creek. Environments, 8, 15. 10.3390/environments8020015

[44] Wang S., Nan Z., Prete D., Ma J., Liao Q., & Zhang Q. (2016). Accumulation, transfer, and potential sources of mercury in the soil-wheat system under field conditions over the Loess Plateau, northwest China. Science of The Total Environmental, 568, 245–252. 10.1016/j.scitotenv.2016.06.034 27300562

[45] Yuan B., & Yue D. (2012). Soil microbial and enzymatic activities across a Chrono sequence of Chinese pine plantation development on the loess plateau of China. Pedosphere, 22, 112. 10.1016/S1002-0160(11)60186-0

[46] Zhang F., Zhidong X., Xu X., Liang L., Chen Z., Dong X., Luo K., Dinis F., & Qiu G. (2022). Terrestrial mercury and methylmercury bioaccumulation and trophic transfer in subtropical urban forest food webs. Chemosphere, 299, 134424. 10.1016/j.chemosphere.2022.134424 35351481

